# Pirh2 mediates the sensitivity of myeloma cells to bortezomib via canonical NF-κB signaling pathway

**DOI:** 10.1007/s13238-017-0500-9

**Published:** 2018-02-13

**Authors:** Li Yang, Jing Chen, Xiaoyan Han, Enfan Zhang, Xi Huang, Xing Guo, Qingxiao Chen, Wenjun Wu, Gaofeng Zheng, Donghua He, Yi Zhao, Yang Yang, Jingsong He, Zhen Cai

**Affiliations:** Multiple Myeloma Treatment Center & Bone Marrow Transplantation Center, The First Affiliated Hospital, College of Medicine, Zhejiang University, Hangzhou, 310003 China

**Keywords:** bortezomib, drug resistance, multiple myeloma, NF-κB, Pirh2

## Abstract

Clinical success of the proteasome inhibitor established bortezomib as one of the most effective drugs in treatment of multiple myeloma (MM). While survival benefit of bortezomib generated new treatment strategies, the primary and secondary resistance of MM cells to bortezomib remains a clinical concern. This study aimed to highlight the role of p53-induced RING-H2 (Pirh2) in the acquisition of bortezomib resistance in MM and to clarify the function and mechanism of action of Pirh2 in MM cell growth and resistance, thereby providing the basis for new therapeutic targets for MM. The proteasome inhibitor bortezomib has been established as one of the most effective drugs for treating MM. We demonstrated that bortezomib resistance in MM cells resulted from a reduction in Pirh2 protein levels. Pirh2 overexpression overcame bortezomib resistance and restored the sensitivity of myeloma cells to bortezomib, while a reduction in Pirh2 levels was correlated with bortezomib resistance. The levels of nuclear factor-kappaB (NF-κB) p65, pp65, pIKBa, and IKKa were higher in bortezomib-resistant cells than those in parental cells. Pirh2 overexpression reduced the levels of pIKBa and IKKa, while the knockdown of Pirh2 via short hairpin RNAs increased the expression of NF-κB p65, pIKBa, and IKKa. Therefore, Pirh2 suppressed the canonical NF-κB signaling pathway by inhibiting the phosphorylation and subsequent degradation of IKBa to overcome acquired bortezomib resistance in MM cells.

## Introduction

The proteasome inhibitor bortezomib is effective at treating multiple myeloma (MM) because the efficacy of bortezomib-based chemotherapy regimens is as high as 80%–90% (Rajkumar, 2016), However, drug resistance limits its repeated use, although the mechanisms are not fully understood (Malard et al., 2017). The mechanism of acquisition of bortezomib resistance and the ways to overcome this resistance are important clinical issues (Chao and Wang, 2016). Hence, the molecular mechanisms of bortezomib resistance need to be urgently explored to enhance the use of existing treatments and to define more effective single or combination therapies. The current established molecular mechanisms underlying resistance to proteasome inhibitors involve constitutive and immunoproteasomes, mutated proteasome subunits, unfolded protein response (UPR) mediators, multidrug efflux transporters, aggresomes, autophagosomes, pro-survival signaling pathway mediators, or bone marrow microenvironmental components (Niewerth et al., 2015). The ubiquitin-proteasome system is the main mechanism controlling protein turnover and maintaining cellular protein homeostasis. E3 ubiquitin ligases determine the specificity of protein degradation, and these ligases have been shown to be closely related to cancer occurrence, development, transfer, and drug resistance (Masumoto and Kitagawa, 2016). Several lines of evidence link E3 ligases with MM and associated drug resistance (Lub et al., 2016).

p53-induced RING-H2 (Pirh2) is a newly discovered E3 ubiquitin ligase induced by p53 activation that has an intrinsic ubiquitin-protein ligase activity for polyubiquitination and subsequent proteasomal degradation. Pirh2 was initially thought to be similar to HDM2, the human counterpart of MDM2 in mice (Halaby et al., 2013). Recent studies have shown that Pirh2 regulates cellular homeostasis in both p53-dependent and p53-independent cellular contexts. Moreover, based on its substrates, the association of Pirh2 with the occurrence and prognosis of malignant tumors has been confirmed (Jung et al., 2012, Daks et al., 2016, Yang et al., 2016). Pirh2 ubiquitinates substrate proteins and directs them through degradation pathways involved in apoptosis induction, cell cycle regulation, and DNA repair. However, the role of Pirh2 in proliferation, invasion, and drug resistance of tumors still needs further investigation. Recently, data from publicly available databases have been used to correlate gene expression in myeloma tumor cells with clinical responses to bortezomib. A reduced expression of Pirh2 was observed in bortezomib-resistant cells using an Affymetrix GeneChip Human Transcriptome Array 2.0 (Huang et al., 2017). These findings helped further elucidating the role of the E3 ligase Pirh2 in the bortezomib resistance of MM, thereby facilitating bortezomib retreatment as a more effective and rational therapeutic strategy in the clinic.

## Results

### Reduced levels of Pirh2 were correlated with bortezomib resistance

**Figure 1:**
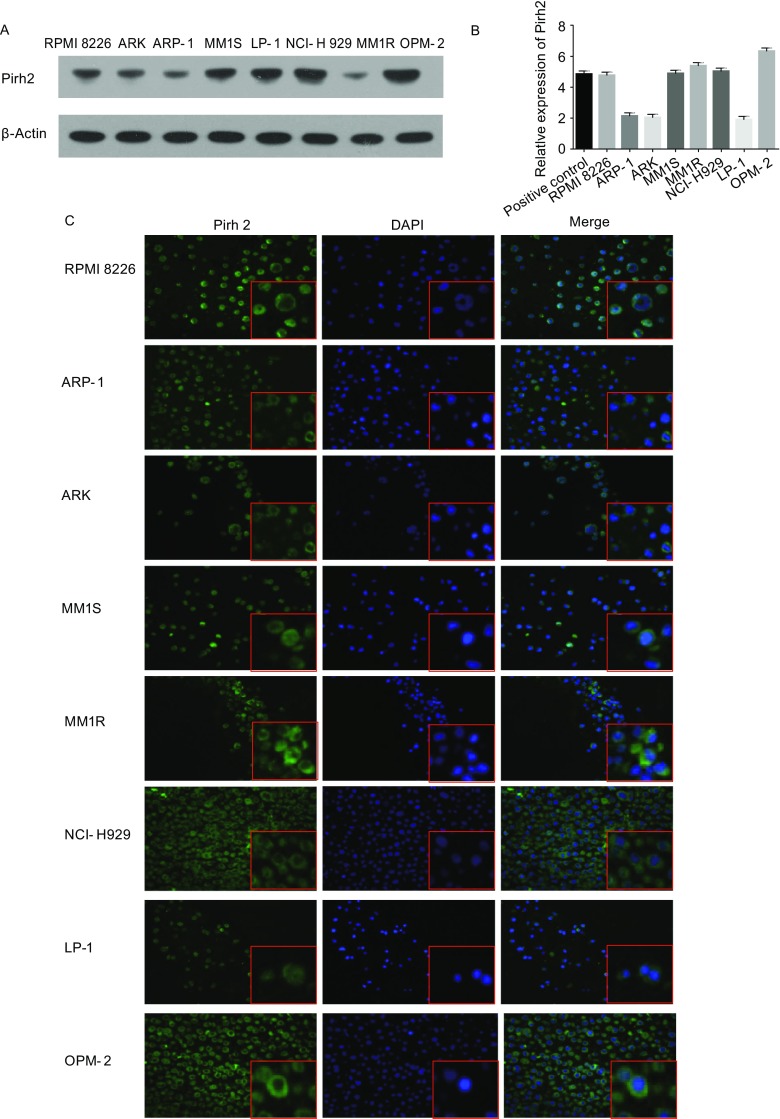
**Pirh2 expression in MM cell lines**. (A) Analysis of Pirh2 expression in human MM cell lines RPMI8226, ARP-1, ARK, MM1S, MM1R, NCI-H929, OPM-2, and LP-1 using Western blotting and (B) The levels of Pirh2 mRNA by qRT-PCR of MM cell lines and MCF-7 cell as positive control. A immunofluorescence showed that Pirh2 in MM cells was expressed mainly in the cytoplasm (C, 400×)

A decreased expression of Pirh2 was observed in the bortezomib-resistant cells RPMI8226.BR and OPM-2.BR and their controls using Affymetrix HTA 2.0. The expression of Pirh2 protein and mRNA was confirmed in the MM cell lines RPMI8226, ARP-1, ARK, MM.1S, MM.1R, NCI-H929, OPM-2, and LP-1. Immunofluorescence revealed that Pirh2 was primarily expressed in the cytoplasm (Fig. 1).

**Figure 2:**
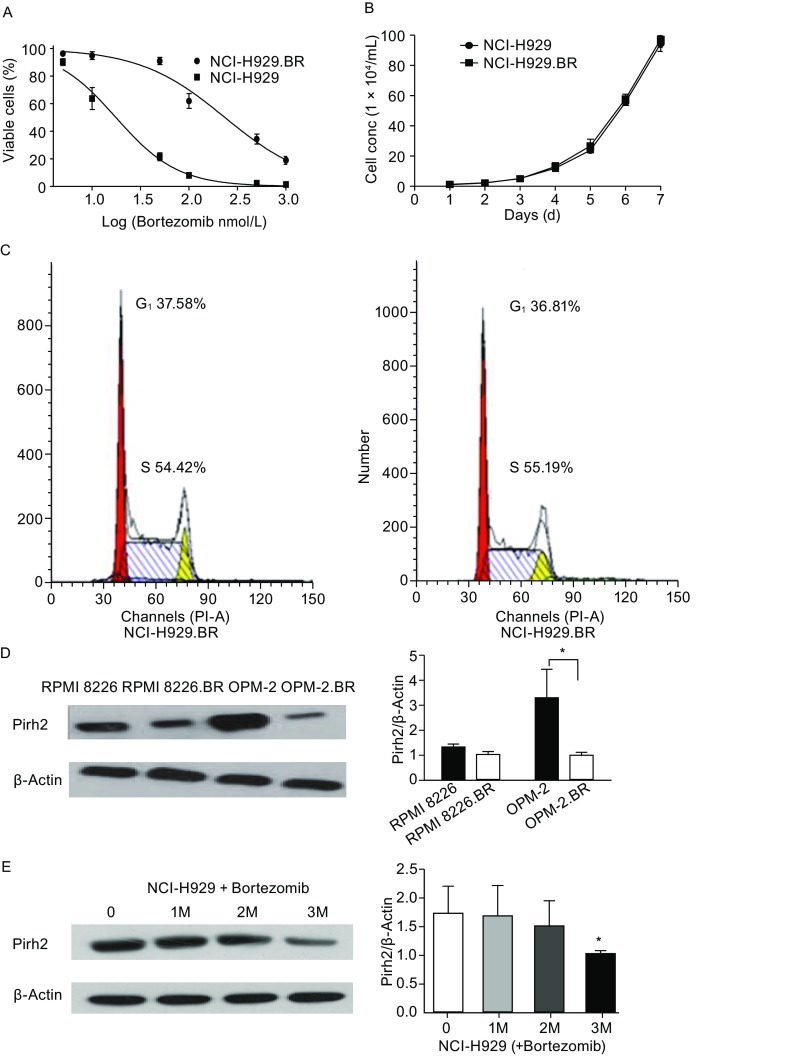
**Establishment of bortezomib-resistant cell line NCI-H929.BR.** (A) CCK-8 assay showed that the IC_50_ of NCI-H929 and NCI-H929.BR treated with bortezomib for 24 h was 17.62 ± 1.92 nmol/L vs. 234.30 ± 6.02 nmol/L; the resistance ratio was 13.30 (*P* < 0.05). (B) Growth curve and (C) flow cytometry results showed no significant difference between the two (*P* > 0.05). (D) Pirh2 expression decreased in bortezomib-resistant cell lines RPMI8226.BR and OPM-2.BR compared with their parental cells. OPM-2.BR shows a corresponding decrease in Pirh2 protein levels compared with OPM-2 (by > 2.0-fold) (*n* = 3). (E) Pirh2 expression levels were found to decline gradually by exposing parental cells NCI-H929 to serially increased drug concentrations for 1–3 months. (*P < 0.05; **P < 0.05, NCI-H929 exposing in bortezomib for 3 months vs. parental cells NCI-H929)

The bortezomib-resistant cell line NCI-H929.BR was established, and changes in Pirh2 expression were identified to evaluate the role of the E3 ubiquitin ligase Pirh2 in bortezomib resistance in MM. As detected with the CCK-8 assay, the IC_50_ of NCI-H929 and NCI-H929.BR cells treated with bortezomib for 24 h was 17.62 ± 1.92 nmol/L and 234.30 ± 6.02 nmol/L, respectively (Fig. 2A); the resistance ratio was 13.30 (*P* < 0.05). Growth curve (Fig. 2B) and flow cytometry results (Fig. 2C) indicated the lack of a significant difference between the bortezomib-resistance and bortezomib-sensitive cells (*P* > 0.05). Compared with that in parental cells, Pirh2 expression was reduced in the bortezomib-resistant cell lines RPMI8226.BR and OPM-2.BR (Fig. 2D). Pirh2 expression levels were also found to gradually decrease over 1–3 months in parental NCI-H929 cells in response to increasing drug concentrations (Fig. 2E).

### Pirh2 was more highly expressed in patients with newly diagnosed MM than in patients with relapsed MM

**Figure 3:**
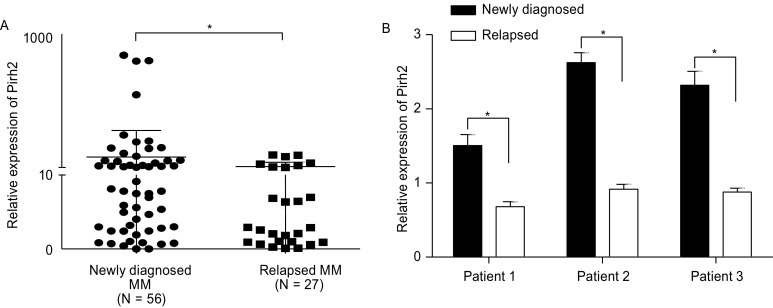
**Pirh2 mRNA expression in primary MM cells**. (A) Pirh2 was more highly expressed in patients with newly diagnosed MM compared with patients with relapsed MM, despite treatment with bortezomib-based therapies. (B) Expression of Pirh2 in CD138^+^ cells decreased in patients with relapsed MM compared with patients with newly diagnosed MM. (*P < 0.05)

Pirh2 mRNA expression was also determined in bone marrow samples obtained from patients with MM. Pirh2 expression was found to be higher in patients with newly diagnosed MM than in patients with relapsed MM treated with bortezomib plus dexamethasone and cyclophosphamide (CTX) (Fig. 3A, *P* < 0.05). Next, CD138^+^ MM cells were isolated from three patients. Pirh2 expression was compared in samples from the same patient at different stages of disease. Pirh2 expression in CD138^+^ cells was lower in patients with relapsed MM than in patients with newly diagnosed MM (Fig. 3B, *P* < 0.05).

### Pirh2 knockdown prevented bortezomib-induced cell apoptosis and antiproliferative effects

**Figure 4:**
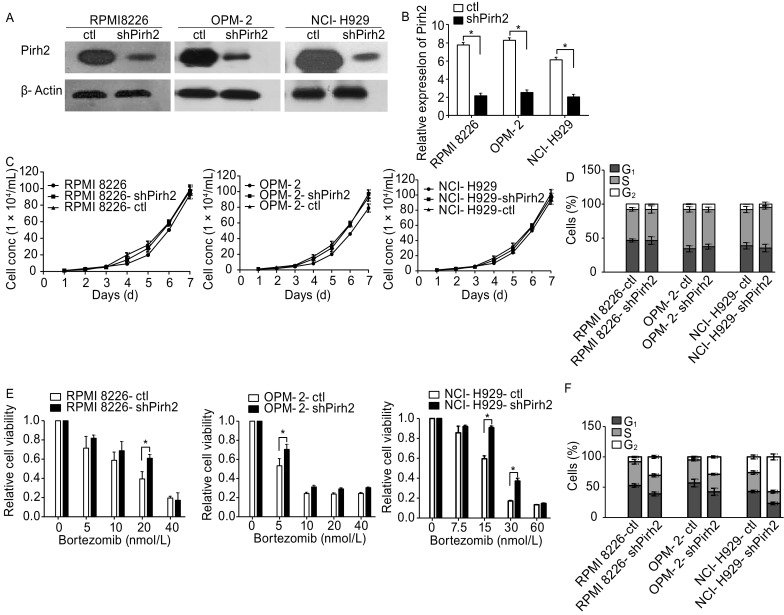

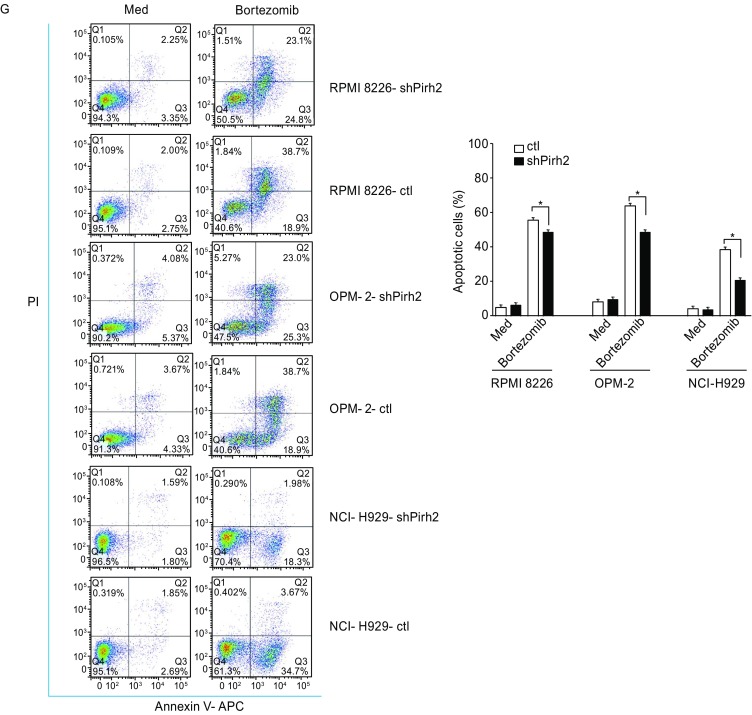
**Characteristics of Pirh2 shRNA cells. Pirh2 knockdown myeloma cell lines RPMI 8226-shPirh2, OPM-2-shPirh2, and NCI-H929-shPirh2 and their controls were established**. (A) Western blot and (B) qRT-PCR were performed to verify transfection efficiency (*P < 0.05). (C) Growth curve using CCK8 and (D) cell cycle analysis by flow cytometry showed no significant difference between cells with Pirh2 knockdown and controls (*P* > 0.05). (E) CCK-8 assay showed that Pirh2 knockdown weakened the inhibition of cell proliferation ability of bortezomib (*P < 0.05). (F) Flow cytometry results showed that the percentages of G_1_ phase in groups with bortezomib treated for RPMI 8226 (10 nmol/L), OPM-2 (10 nmol/L), and NCI-H929 (15 nmol/L) were as follows: RPMI 8226-shPirh2 vs. RPMI 8226-ctl, 39.03% ± 3.20% vs. 52.84% ± 42.89%; OPM-2-shPirh2 vs. OPM-2-ctl, 42.40% ± 5.84% vs. 57.00% ± 6.23%; NCI-H929-shPirh2 vs. NCI-H929-ctl, 23.37% ± 2.12% vs. 42.91% ± 1.89%. Pirh2 shRNA prevented bortezomib-induced cell apoptosis. (G) The percentage of apoptotic cells in groups treated with bortezomib or Med for 24 h and the results of experiments repeated three times for RPMI 8226 (10 nmol/L), OPM-2 (10 nmol/L), and NCI-H929 (15 nmol/L)

Western blotting and qRT-PCR were performed to verify transfection efficiency in the myeloma cell lines RPMI8226-shPirh2, OPM-2-shPirh2, NCI-H929-shPirh2 and their corresponding controls (Fig. 4A and 4B). Growth curve and cell cycle analysis demonstrated the lack of significant difference between cells with Pirh2 knockdown and controls without bortezomib treatment (Fig. 4C and 4D, *P* > 0.05). However, Pirh2 knockdown enabled the transition of MM cells from G_1_ phase to S and G_2_ phases in the presence of bortezomib (Fig. 4D) and weakened the inhibition of cell proliferation by bortezomib (Fig. 4E). The percentage of cells in G_1_ phase in various groups was as follows: RPMI8226-shPirh2 vs. RPMI8226-ctl, 39.03% ± 3.20% vs. 52.84% ± 42.89%; OPM-2-shPirh2 vs. OPM-2-ctl, 42.40% ± 5.84% vs. 57.00% ± 6.23%; and NCI-H929-shPirh2 vs. NCI-H929-ctl, 23.37% ± 2.12% vs. 42.91% ± 1.89% (Fig. 4F, *P* < 0.05). In addition, Pirh2 knockdown reduced bortezomib-induced apoptosis in MM cells. The percentage of apoptotic cells in various groups was as follows: RPMI8226-shPirh2 vs. RPMI8226-ctl, 47.90% ± 1.63% vs. 55.60% ± 2.86%; OPM-2-shPirh2 vs. OPM-2-ctl, 48.30% ± 1.17% vs. 63.60% ± 1.24%; and NCI-H929-shPirh2 vs. NCI-H929-ctl, 20.28% ± 0.98% vs. 38.37% ± 1.34% (Fig. 4G, *P* < 0.05).

### Pirh2 overexpression enhanced bortezomib-induced cell apoptosis and antiproliferative effects and resulted in G_1_ phase cell cycle arrest in MM cells

**Figure 5:**
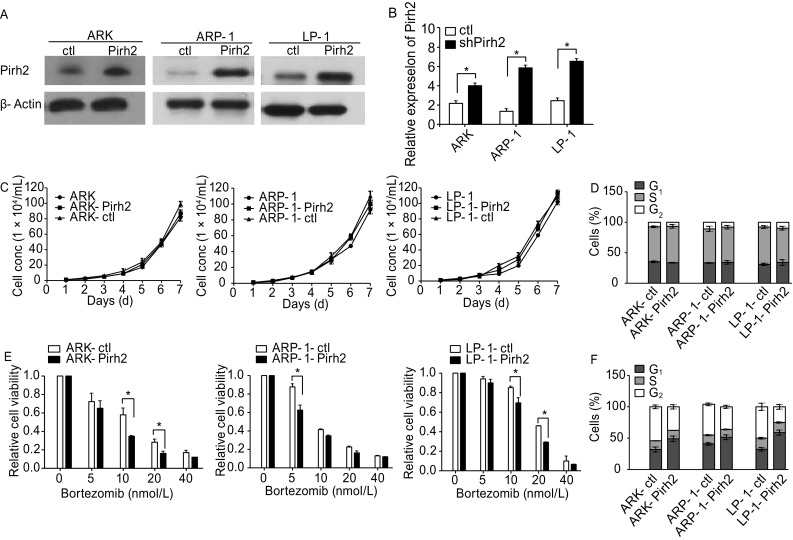

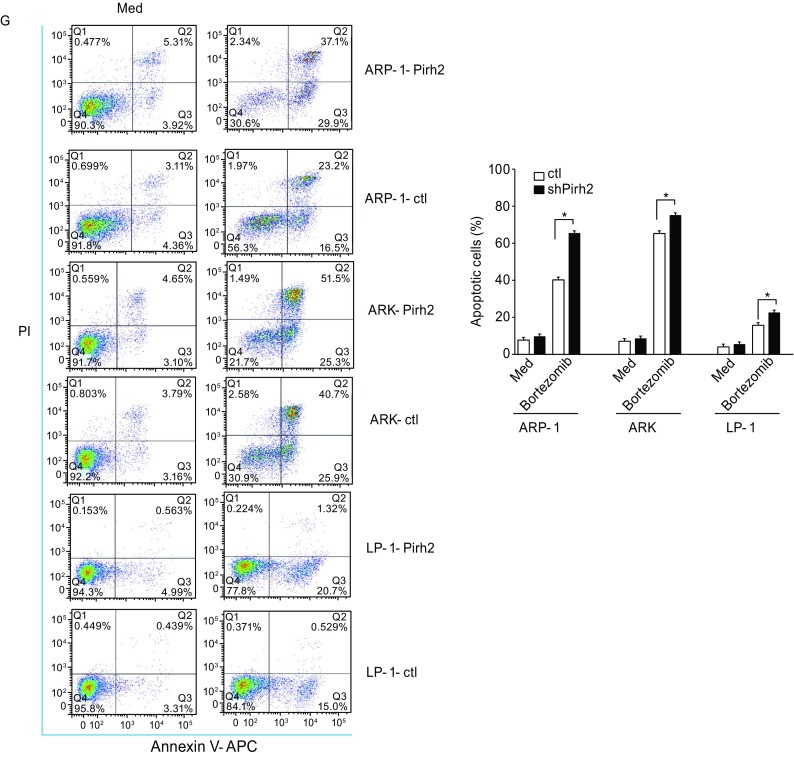
**Characteristics of Pirh2-overexpression cells**. Pirh2-overexpression myeloma cell lines ARP-1-Pirh2, ARK-Pirh2, and LP-1-Pirh2 and their controls were established. (A) Western blot and (B) qRT-PCR were performed to verify transfection efficiency (*P < 0.05). (C) Growth curve using CCK8 and (D) cell cycle analysis by flow cytometry showed no significant difference between cells with Pirh2 overexpression and controls (*P* > 0.05). (E) CCK-8 assay showed that Pirh2 overexpression increased the inhibition of cell proliferation ability of bortezomib (*P < 0.05). (F) Pirh2 overexpression made MM cell cycle arrest in G_1_ phase with bortezomib treated for ARP-1 (10 nmol/L), ARK (10 nmol/L), and LP-1 (10 nmol/L). The percentages of G_1_ phase in groups were as follows: ARP-1-Pirh2 vs. ARP-1-ctl, 51.56% ± 3.91% vs. 40.88% ± 2.09%; ARK-Pirh2 vs. ARK-ctl, 49.10% ± 4.32% vs. 31.90% ± 3.98%; LP-1-Pirh2 vs. LP-1-ctl, 58.90% ± 4.06% vs. 32.40% ± 2.76% (*P* < 0.05). (G) Pirh2 overexpression sensitized bortezomib-induced cell apoptosis. The percentage of apoptosis cells in groups treated with bortezomib or Med for 24 h and the results of experiments repeated three times for ARP-1 (10 nmol/L), ARK (10 nmol/L), and LP-1 (10 nmol/L) (*P < 0.05)

Pirh2-overexpressing myeloma cell lines ARP-1-Pirh2, ARK-Pirh2, LP-1-Pirh2 and their corresponding controls were established as described earlier. Western blotting and qRT-PCR were performed to verify transfection efficiency (Fig. 5A and 5B). Growth curve and cell cycle analysis demonstrated the lack of significant difference between Pirh2-overexpressing cells and controls (Fig. 5C and 5D, *P* > 0.05). However, Pirh2 overexpression increased the inhibition of cell proliferation by bortezomib (Fig. 5E, *P* < 0.05) and arrested MM cells in G_1_ phase. The percentage of cells in G_1_ phase in various groups was as follows: ARP-1-Pirh2 vs. ARP-1-ctl, 51.56% ± 3.91% vs. 40.88% ± 2.09%; ARK-Pirh2 vs. ARK-ctl, 49.10% ± 4.32% vs. 31.90% ± 3.98%; and LP-1-Pirh2 vs. LP-1-ctl, 58.90% ± 4.06% vs. 32.40% ± 2.76% (Fig. 5F, *P* < 0.05) and Furthermore, Pirh2 overexpression increased bortezomib-induced apoptosis in MM cells. The percentage of apoptotic cells in various groups was as follows: ARP-1-Pirh2 vs. ARP-1-ctl, 66.90% ± 3.73% vs. 41.70% ± 1.86%; ARK-Pirh2 vs. ARK-ctl, 76.80% ± 4.17% vs. 66.60% ± 3.24%; and LP-1-Pirh2 vs. LP-1-ctl, 22.02% ± 1.23% vs. 15.53% ± 2.03% (Fig. 5G, *P* < 0.05).

### Pirh2 mediated the sensitivity of myeloma cells to bortezomib but not to CTX or melphalan

**Figure 6:**
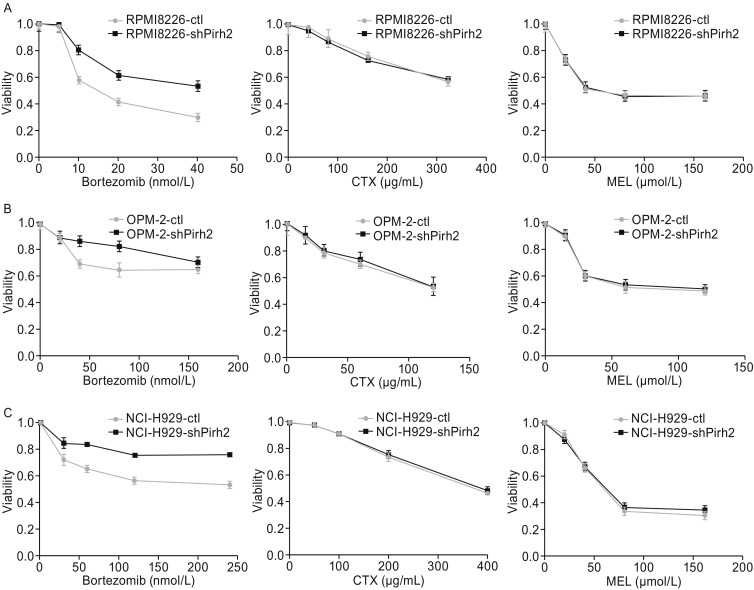
**Pirh2 mediated the sensitivity of myeloma cells to bortezomib but not to CTX and Mel**. The CCK-8 assay showed that Pirh2 knockdown weakened the inhibition of cell proliferation ability of bortezomib (*P* < 0.05) but did not affect the antiproliferative effect of CTX and Mel (*P* > 0.05) in cell lines RPMI 8226-shPirh2 (A), OPM-2-shPirh2 (B), and NCI-H929-shPirh2 (C).

The CCK-8 assay demonstrated that Pirh2 knockdown weakened the inhibition of cell proliferation by bortezomib (Fig. 6, *P* < 0.05) but did not affect the antiproliferative effect of CTX or melphalan (Mel) on the cell lines RPMI8226-shPirh2, OPM-2-shPirh2, and NCI-H929-shPirh2 (Fig. 6, *P* > 0.05).

### Pirh2 suppressed the nuclear factor-kappaB (NF-κB) signaling pathway in bortezomib-resistant myeloma cells

**Figure 7:**
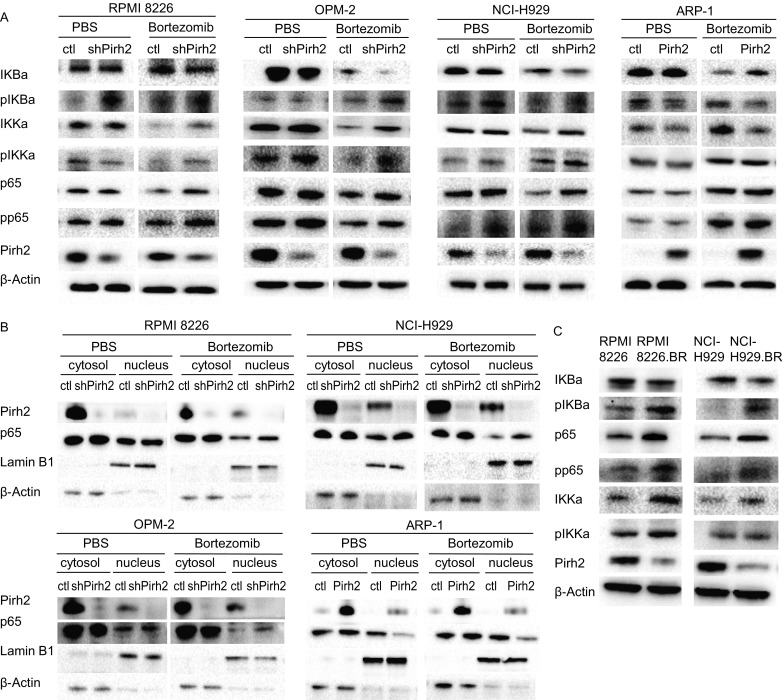
**Basal protein levels of the NF-κB signaling pathway**. Pirh2 overexpression markedly decreased the content of pIKBa and IKKa; Pirh2 shRNA increased the expression of NF-κB p65, pIKBa, and IKKa (A). Pirh2 decreased NF-κB subunit p65 nuclear protein levels and Pirh2 shRNA increased NF-κB p65 protein levels in the nucleus (B). Higher NF-KB p65, pp65, pIKBa, and IKKa protein levels were observed in the bortezomib-resistant cells RPMI 8226.BR and NCI-H929.BR compared with the nucleus of parent cells (C)

Increased nuclear factor-kappaB (NF-κB) expression has been observed in patients with refractory primary myeloma, which is thought to be linked to drug resistance (Turner et al., 2016). Thus, we investigated whether Pirh2 overcomes bortezomib resistance in myeloma cells by inhibiting the NF-κB pathway. The degradation of IKBa and the release of NF-κB subunits were preceded by the phosphorylation of IKBa. Additionally, Pirh2 overexpression markedly decreased the levels of pIKBa and IKKa, while Pirh2 knockdown via shRNA increased the expression of NF-κB p65, pIKBa, and IKKa (Fig. 7A). Pirh2 overexpression decreased the nuclear protein levels of NF-κB subunit p65, whereas Pirh2 knockdown exerted the opposite effect (Fig. 7B). Higher protein levels of NF-κB p65, pp65, pIKBa, and IKKa were observed in the nuclei of bortezomib-resistant cells RPMI8226.BR and NCI-H929.BR than in the nuclei of the parental cells (Fig. 7C).

**Figure 8:**
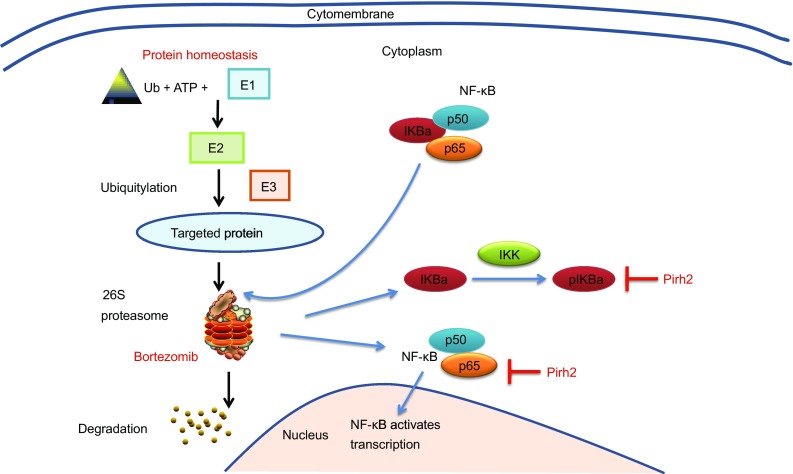
**Pirh2 mediate the sensitivity of myeloma cells to bortezomib via the canonical NF-κB signaling pathway**

Therefore, Pirh2 likely mediates the sensitivity of myeloma cells to bortezomib via the canonical NF-κB signaling pathway (Fig. 8).

## Discussion

The proteasome inhibitor bortezomib is usually effective against MM. However, drug resistance limits its repeated use, although the mechanisms are not fully understood. The ubiquitin-proteasome pathway (UPP) plays a crucial role in maintaining steady-state protein levels and regulating many biological processes. Increasing evidence indicates that the UPP plays an important role in oncogenesis, cancer development, disease progression, and chemoresistance (Cao and Mao, 2011; Tu et al., 2012; Micel et al., 2013; Yerlikaya and Yontem, 2013; Gandhi et al., 2014; Lu et al., 2014; Brinkmann et al., 2015; Wu et al., 2015). For example, the cellular proteasome is an important molecular target in MM therapy, clinical trial development, and the application of proteasome inhibitors. Therefore, inhibiting the UPP may be an effective approach to treat MM (Landis-Piwowar, 2012). E3 ligases carry out the final step in the ubiquitination cascade and determine the substrate protein to be ubiquitylated before mediating the transfer of ubiquitin residues from an E2 enzyme to a lysine on the target. Thus, E3 ligases are of interest as drug targets because of their ability to regulate protein stability and functions (Liu et al., 2014), especially in oncogenesis (Jung et al., 2012, Hsieh et al., 2013, Severe et al., 2013, Sharma and Nag, 2014, Zhang et al., 2014, Hao and Huang, 2015, Yin et al., 2015), cancer progression (Lou and Wang, 2014, Sun and Denko, 2014, Goka and Lippman, 2015), metastasis (Wang et al., 2012), disease prognosis (Bielskiene et al., 2015, Hou and Deng, 2015) and chemotherapy resistance (Nelson et al., 2016, Petzold et al., 2016). A growing number of E3 ligases and their substrate proteins have emerged as crucial players in drug resistance, and new insights have been obtained into the roles of E3 ubiquitin ligases in bortezomib resistance (Malek et al., 2016). Jones and his colleagues (Jones et al., 2012) have found that the E3 ubiquitin ligase HDM2 played crucial roles in the cross-resistance of the myeloma cell line NCI-H929 to bortezomib, doxorubicin, cisplatin, and Mel. Researchers have also found that HDM2 inhibition by bortezomib can enhance cellular sensitivity to bortezomib and overcome bortezomib resistance (Ooi et al., 2009). Meanwhile, Chauhan and colleagues indicated that the inhibitor of the deubiquitylating enzyme USP7 could induce apoptosis in myeloma cells that are resistant to conventional therapies, including bortezomib, by inhibiting HDM2 and p21 (Chauhan et al., 2012).

The E3 ligase Pirh2 regulates the turnover and function of proteins involved in cell proliferation and differentiation, cell cycle checkpoint regulation, and cell death (Halaby et al., 2013). Previous studies have demonstrated the role of Pirh2 as a tumor suppressor and prognostic marker in various human cancer subtypes (Hakem et al., 2011, Halaby et al., 2013). However, human Pirh2 also has been reported to be overexpressed in cancers, such as breast cancer, and was found to be highly associated with tumor grade, size, and Ki-67 expression (Yang et al., 2016).

The current study reported the role of Pirh2 in MM, especially in the resistance of myeloma cells to bortezomib. Pirh2 expression was observed to be reduced in bortezomib-resistant cells. A bortezomib-resistant cell line NCI-H929.BR was established to further evaluate the role of Pirh2 in bortezomib resistance in MM. The Pirh2 expression levels gradually decreased in parental cells in response to increasing drug concentrations over several months. We also observed higher expression of Pirh2 in patients with newly diagnosed MM than in patients with relapsed MM treated with bortezomib-based therapies, indicating that MM patients with low Pirh2 levels were refractory to bortezomib. Pirh2 knockdown via shRNA prevented bortezomib-induced cell apoptosis and antiproliferative effects, whereas Pirh2 overexpression exerted the opposite effects and arrested cells MM cells in G_1_ phase. Notably, Pirh2 knockdown or overexpression in the absence of bortezomib treatment did not affect cell proliferation, apoptosis, or cell cycle distribution, and Pirh2 mediated the sensitivity of myeloma cells to bortezomib but not to Mel, CTX, or dexamethasone (data not shown). Therefore, reduction in Pirh2 levels was associated with resistance to bortezomib, suggesting the possible involvement of Pirh2 in the acquisition of bortezomib resistance in MM.

We also explored the ability of Pirh2 to negatively regulate the NF-κB pathway in MM cells. Proteasome inhibition by bortezomib ultimately results in UPR-mediated apoptosis due to the accumulation of unprocessed and misfolded proteins. Signaling through canonical or noncanonical pathways leads to the phosphorylation, ubiquitination, and subsequent degradation of IkB kinases through the proteasome pathway. This results in the translocation of NF-κB to the nucleus and transcription of target genes that are associated with mechanisms of resistance, such as the acquisition of mutations in the 26S proteasome, overexpression of the PSMB5 subunit, upregulation of heat shock proteins, or increased activity of the aggresome pathway (Kumar and Rajkumar, 2008). The present study highlighted a molecular mechanism by which Pirh2 overcame bortezomib resistance in myeloma cells. NF-κB is activated in patients with bortezomib-refractory primary MM and bortezomib-resistant MM, which is associated with the increased basal nuclear localization of NF-κB p65 (Raninga et al., 2016). Increased NF-κB p65, pp65, pIKBa, and IKKa levels were observed in bortezomib-resistant cells. Pirh2 overexpression reduced the levels of pIKBa and IKKa, while Pirh2 knockdown increased the expression of NF-κB p65, pIKBa, and IKKa. Therefore, Pirh2 suppressed the canonical NF-κB signaling pathway by inhibiting the phosphorylation and subsequent degradation of IKBa and thus overcame acquired bortezomib resistance in myeloma cells. However, whether Pirh2 regulates the NF-κB signaling pathway in MM via other mediators or mechanisms warrants further investigation.

In conclusion, the inhibition of Pirh2 expression is associated with the acquisition of bortezomib resistance in myeloma cells. Moreover, Pirh2 overexpression overcomes bortezomib resistance in myeloma cells by inhibiting the NF-κB signaling pathway. Therefore, the ability of the E3 ligase Pirh2 to negatively regulate the NF-κB signaling pathway and promote malignant phenotypes highlights the importance of this novel tumor suppressor in MM and the necessity for its regulation. Pirh2-promoting drugs may thus be considered for single-agent or combination therapies to circumvent bortezomib resistance in MM and improve survival outcomes in patients with MM.

## Materials and methods

### Cells and reagents

Human MM cell lines RPMI8226, MM.1S, MM.1R, ARP-1, ARK, NCI-H929, LP-1, and OPM-2 and bortezomib-resistant cell lines RPMI8226.BR and OPM-2.BR were provided by Dr. Qing Yi (Department of Cancer Biology, Lerner Research Institute, Cleveland Clinic, OH, USA). The bortezomib-resistant cell line NCI-H929.BR was developed by exposing parental cells to sublethal concentrations of bortezomib. Primary CD138^+^ cells from the bone marrow of MM patients and peripheral blood mononuclear cells from healthy individuals were obtained after approval from the ethics committee of the First Affiliated Hospital, Zhejiang University School of Medicine, China, and informed consent was obtained from the participants. CD138^+^ cells were collected using positive selection with CD138 microbeads (Miltenyi Biotech, CA, USA). Dimethyl sulfoxide and propidium iodide (PI) were procured from Sigma-Aldrich (MO, USA). Bortezomib was obtained from Millennium Pharmaceuticals, Inc. (MA, USA). The Annexin V Apoptosis Detection Kit and Fluorescein Isothiocyanate (FITC)/PI were purchased from eBioscience (CA, USA). Primary antibodies against IKBa, pIKBa, p65, pp65, IKKa, and pIKKa were procured from Cell Signaling Technology (MA, USA). Primary antibodies against Pirh2 [EPR14980 and 1H10] were purchased from Abcam. β-Actin was obtained from Sigma-Aldrich (MO, USA). Horseradish peroxidase-conjugated anti-mouse and anti-rabbit antibodies were procured from Jackson ImmunoResearch Laboratories (PA, USA).

### Cell culture

Human MM cell lines RPMI8226, MM.1S, MM.1R, ARP-1, ARK, NCI-H929, LP-1, and OPM-2; bortezomib-resistant cell lines RPMI8226.BR, OPM-2.BR, and NCI-H929.BR; and primary cells were all cultured in RPMI 1640 medium (Thermo Scientific, HyClone) supplemented with 10% fetal bovine serum (Thermo Scientific, HyClone) and 1% L-glutamine at 37°C in a humidified atmosphere with 5% CO_2_.

### Establishment of bortezomib-resistant cell lines

The cell line NCI-H929.BR was generated by exposing parental NCI-H929 cells to sublethal concentrations of bortezomib for 6 months as reported (Zhu et al., 2009). Bortezomib resistance was confirmed by measuring cell proliferation in response to bortezomib exposure.

### Cell proliferation assay

The cell counting kit 8 (CCK-8) assay was used to assess MM cell proliferation. A total of 1–2 × 10^4^/well MM cells were seeded in 96-well plates and incubated at 37°C in a humidified atmosphere with 5% CO_2_. Each cell line was assessed in triplicate. CCK-8 solution (10 μL) was added into each well 2 h before measuring the absorbance. Absorbance at 450 nm was measured using the ELX800 microplate reader (BioTek, USA).

### Cell cycle analysis

Cells were cultured (5 × 10^5^/well) with or without 10 mmol/L bortezomib in 6-well plates for 24 h. Then, the cells were collected and permeabilized with precooled 75% ethanol at 4°C overnight. The next day, the cells were washed with phosphate-buffered saline (PBS) and incubated with 0.01% RNase A for 30 min at 37°C. Next, the cells were incubated with 0.5% PI in the dark for 30 min. DNA content was measured by flow cytometry (BD Biosciences, CA, USA). The data were analyzed using ModFit software (version 3.2, Verity Software House).

### Assessment of apoptosis

MM cells (2 × 10^5^/mL) were cultured in 24-well plates at 37°C in a humidified atmosphere with 5% CO_2_ for 24 h with or without bortezomib to identify apoptotic cells. The cells were then harvested, washed twice with PBS, resuspended in 200–300 μL of staining buffer, and stained with Annexin V-FITC/PI according to the manufacturer’s instructions. The cells were subjected to flow cytometry, and the data were analyzed using FlowJo version 7.6.1.

### Western blot analysis

Cells were collected and extracted with lysis buffer to detect changes in cellular protein levels. Supernatants containing total cellular protein were collected for Western blotting. Equal amounts of proteins (40–60 μg, depending on the protein) were separated by 8%–12% sodium dodecyl sulfate-polyacrylamide gel electrophoresis and transferred onto polyvinylidene difluoride membranes (Merck Millipore, Germany). The membranes were incubated with the corresponding primary antibodies overnight at 4°C after blocking with 5% non-fat milk. The membranes were then washed with Tris-buffered saline with Tween 20 (TBST) and incubated with HRP-conjugated anti-rabbit or anti-mouse antibodies in TBST at room temperature for 2 h. The membranes were again washed with TBST, and protein bands were detected on an X-ray film using an enhanced chemiluminescence detection kit for HRP (Biological Industries Israel, Beit Haemek Ltd., Israel).

### RNA extraction and quantitative real-time polymerase chain reaction

Total RNA isolation from cells and cDNA synthesis were performed using Trizol and a Reverse Transcriptase kit (Takara, Otsu, Japan), respectively, according to the manufacturer’s instructions. Semiquantitative real-time polymerase chain reaction (qRT-PCR) was performed with SYBR Premix Ex Taq II (TliRNaseH Plus) (Takara) in combination with the iQ5 Multicolor Real-Time PCR Detection System (Bio-Rad Inc.) according to the manufacturer’s instructions. cDNA encoding the indicated genes was amplified with the following specific primers: Pirh2 forward, 5′TGCAATCACTCGTTTATGCTGTCTA3′ and reverse, 5′ACCCTGGGTACCGAAGCCTA3′; and GAPDH forward, 5′GCTGGTGGTCCAGGGGTCTTACT3′ and reverse, 5′TCAACGACCACTTTGTCAAGCTCA3′.

### Generation of Pirh2-targeting short hairpin RNA and Pirh2-overexpressing myeloma cell lines

Because Pirh2 expression was reduced in bortezomib-resistant cell lines, we investigated whether Pirh2 facilitates bortezomib-induced apoptosis in myeloma cell lines. Hence, Pirh2 knockdown myeloma cell lines and short hairpin RNA (shRNA) sequences for targeting Pirh2 mRNA (shPirh, 5′GCACAGACTCCTATGCCATCA3′) and scrambled shRNA oligos (used as a negative control; 5′TTCTCCGAACGTGTCACGT3′) were generated. All shRNAs were designed and synthesized by Shanghai GenePharma Co., Ltd. (Shanghai, China). The resultant cells infected with lentiviruses containing Pirh2-targeting shRNA sequences were referred to as RPMI8226-shPirh2, OPM-2-shPirh2, and NCI-H929-shPirh2, and the cells infected with the corresponding controls were referred to as RPMI8226-ctl, OPM-2-ctl, and NCI-H929-ctl.

For the stable overexpression of Pirh2, cells were infected with lentiviral particles harboring the Pirh2 expression vector LV5-EF1a-GFP/Puro-Pirh2 with full-length cDNA sequence from GenBank (Accession Number: BC047393; purchased from GenePharma, Shanghai, China). The Pirh2-overexpressing myeloma cell lines were named ARP-1-Pirh2, ARK-Pirh2 and LP-1-Pirh2, and their corresponding controls were named ARP-1-ctl, ARK-ctl and LP-1-ctl. Target cells were infected with lentiviruses for 24–48 h. The optimal infection efficacy was determined by assaying different multiplicity of infections with or without 2 μg/mL puromycin according to the manufacturer’s protocol.

### Statistical analysis

Data were presented as the mean ± standard deviation. Two-tailed Student’s *t* test was used to determine significant differences between two groups, and one-way analysis of variance was used to estimate differences between three or more groups. *P* values lower than 0.05 were considered significant. All analyses were performed using GraphPad Prism 5.0 (GraphPad Software, CA, USA).

## References

[CR1] Bielskiene K, Bagdoniene L, Mozuraitiene J, Kazbariene B, Janulionis E (2015). E3 ubiquitin ligases as drug targets and prognostic biomarkers in melanoma. Medicina (Kaunas).

[CR2] Brinkmann K, Schell M, Hoppe T, Kashkar H (2015). Regulation of the DNA damage response by ubiquitin conjugation. Front Genet.

[CR3] Cao B, Mao X (2011). The ubiquitin-proteasomal system is critical for multiple myeloma: implications in drug discovery. Am J Blood Res.

[CR4] Chao A, Wang TH (2016). Molecular mechanisms for synergistic effect of proteasome inhibitors with platinum-based therapy in solid tumors. Taiwan J Obstet Gynecol.

[CR5] Chauhan D, Tian Z, Nicholson B, Kumar KG, Zhou B, Carrasco R, McDermott JL, Leach CA, Fulcinniti M, Kodrasov MP (2012). A small molecule inhibitor of ubiquitin-specific protease-7 induces apoptosis in multiple myeloma cells and overcomes bortezomib resistance. Cancer Cell.

[CR6] Daks A, Petukhov A, Fedorova O, Shuvalov O, Merkulov V, Vasileva E, Antonov A, Barlev NA (2016). E3 ubiquitin ligase Pirh2 enhances tumorigenic properties of human non-small cell lung carcinoma cells. Genes Cancer.

[CR7] Gandhi AK, Kang J, Havens CG, Conklin T, Ning Y, Wu L, Ito T, Ando H, Waldman MF, Thakurta A (2014). Immunomodulatory agents lenalidomide and pomalidomide co-stimulate T cells by inducing degradation of T cell repressors Ikaros and Aiolos via modulation of the E3 ubiquitin ligase complex CRL4(CRBN.). Br J Haematol.

[CR8] Goka ET, Lippman ME (2015). Loss of the E3 ubiquitin ligase HACE1 results in enhanced Rac1 signaling contributing to breast cancer progression. Oncogene.

[CR9] Hakem A, Bohgaki M, Lemmers B, Tai E, Salmena L, Matysiak-Zablocki E, Jung YS, Karaskova J, Kaustov L, Duan S (2011). Role of Pirh2 in mediating the regulation of p53 and c-Myc. PLoS Genet.

[CR10] Halaby MJ, Hakem R, Hakem A (2013). Pirh2: an E3 ligase with central roles in the regulation of cell cycle, DNA damage response, and differentiation. Cell Cycle.

[CR11] Hao Z, Huang S (2015). E3 ubiquitin ligase Skp2 as an attractive target in cancer therapy. Front Biosci (Landmark Ed).

[CR12] Hou YC, Deng JY (2015). Role of E3 ubiquitin ligases in gastric cancer. World J Gastroenterol.

[CR13] Hsieh SC, Kuo SN, Zheng YH, Tsai MH, Lin YS, Lin JH (2013). The E3 ubiquitin ligase SIAH2 is a prosurvival factor overexpressed in oral cancer. Anticancer Res.

[CR14] Huang YH, Li SC, Huang LH, Chen PC, Lin YY, Lin CC, Kuo HC (2017). Identifying genetic hypomethylation and upregulation of toll-like receptors in Kawasaki disease. Oncotarget..

[CR15] Jones RJ, Bjorklund CC, Baladandayuthapani V, Kuhn DJ, Orlowski RZ (2012). Drug resistance to inhibitors of the human double minute-2 E3 ligase is mediated by point mutations of p53, but can be overcome with the p53 targeting agent RITA. Mol Cancer Ther.

[CR16] Jung YS, Qian Y, Chen X (2012). Pirh2 RING-finger E3 ubiquitin ligase: its role in tumorigenesis and cancer therapy. FEBS Lett.

[CR17] Kumar S, Rajkumar SV (2008). Many facets of bortezomib resistance/susceptibility. Blood.

[CR18] Landis-Piwowar KR (2012). Proteasome inhibitors in cancer therapy: a novel approach to a ubiquitous problem. Clin Lab Sci.

[CR19] Liu J, Shaik S, Dai X, Wu Q, Zhou X, Wang Z, Wei W (2014). Targeting the ubiquitin pathway for cancer treatment. Biochim Biophys Acta.

[CR20] Lou Z, Wang S (2014). E3 ubiquitin ligases and human papillomavirus-induced carcinogenesis. J Int Med Res.

[CR21] Lu G, Middleton RE, Sun H, Naniong M, Ott CJ, Mitsiades CS, Wong KK, Bradner JE, Kaelin WG (2014). The myeloma drug lenalidomide promotes the cereblon-dependent destruction of Ikaros proteins. Science.

[CR22] Lub S, Maes K, Menu E, De Bruyne E, Vanderkerken K, Van Valckenborgh E (2016). Novel strategies to target the ubiquitin proteasome system in multiple myeloma. Oncotarget.

[CR23] Malard F, Harousseau JL, Mohty M (2017). Multiple myeloma treatment at relapse after autologous stem cell transplantation: a practical analysis. Cancer Treat Rev.

[CR24] Malek E, Abdel-Malek MA, Jagannathan S, Vad N, Karns R, Jegga AG, Broyl A, van Duin M, Sonneveld P, Cottini F (2016). Pharmacogenomics and chemical library screens reveal a novel SCFSKP2 inhibitor that overcomes Bortezomib resistance in multiple myeloma. Leukemia..

[CR25] Masumoto K, Kitagawa M (2016). E3 ubiquitin ligases as molecular targets in human oral cancers. Curr Cancer Drug Targets.

[CR26] Micel LN, Tentler JJ, Smith PG, Eckhardt GS (2013). Role of ubiquitin ligases and the proteasome in oncogenesis: novel targets for anticancer therapies. J Clin Oncol.

[CR27] Nelson JK, Cook EC, Loregger A, Hoeksema MA, Scheij S, Kovacevic I, Hordijk PL, Ovaa H, Zelcer N (2016). Deubiquitylase inhibition reveals liver X receptor-independent transcriptional regulation of the E3 Ubiquitin Ligase IDOL and lipoprotein uptake. J Biol Chem.

[CR28] Niewerth D, Jansen G, Assaraf YG, Zweegman S, Kaspers GJ, Cloos J (2015). Molecular basis of resistance to proteasome inhibitors in hematological malignancies. Drug Resist Updat.

[CR29] Ooi MG, Hayden PJ, Kotoula V, McMillin DW, Charalambous E, Daskalaki E, Raje NS, Munshi NC, Chauhan D, Hideshima T (2009). Interactions of the Hdm2/p53 and proteasome pathways may enhance the antitumor activity of bortezomib. Clin Cancer Res.

[CR30] Petzold G, Fischer ES, Thoma NH (2016). Structural basis of lenalidomide-induced CK1alpha degradation by the CRL4 ubiquitin ligase. Nature..

[CR31] Rajkumar SV (2016). Myeloma today: disease definitions and treatment advances. Am J Hematol.

[CR32] Raninga PV, Di Trapani G, Vuckovic S, Tonissen KF (2016). TrxR1 inhibition overcomes both hypoxia-induced and acquired bortezomib resistance in multiple myeloma through NF-small ka, Cyrillicbeta inhibition. Cell Cycle.

[CR33] Severe N, Dieudonne FX, Marie PJ (2013). E3 ubiquitin ligase-mediated regulation of bone formation and tumorigenesis. Cell Death Dis.

[CR34] Sharma P, Nag A (2014). CUL4A ubiquitin ligase: a promising drug target for cancer and other human diseases. Open Biol.

[CR35] Sun RC, Denko NC (2014). Hypoxic regulation of glutamine metabolism through HIF1 and SIAH2 supports lipid synthesis that is necessary for tumor growth. Cell Metab.

[CR36] Tu Y, Chen C, Pan J, Xu J, Zhou ZG, Wang CY (2012). The Ubiquitin Proteasome Pathway (UPP) in the regulation of cell cycle control and DNA damage repair and its implication in tumorigenesis. Int J Clin Exp Pathol.

[CR37] Turner JG, Kashyap T, Dawson JL, Gomez J, Bauer AA, Grant S, Dai Y, Shain KH, Meads M, Landesman Y, Sullivan DM (2016). XPO1 inhibitor combination therapy with bortezomib or carfilzomib induces nuclear localization of IkappaBalpha and overcomes acquired proteasome inhibitor resistance in human multiple myeloma. Oncotarget.

[CR38] Wang G, Chan CH, Gao Y, Lin HK (2012). Novel roles of Skp2 E3 ligase in cellular senescence, cancer progression, and metastasis. Chin J Cancer.

[CR39] Wu B, Chu X, Feng C, Hou J, Fan H, Liu N, Li C, Kong X, Ye X, Meng S (2015). Heat shock protein gp96 decreases p53 stability by regulating Mdm2 E3 ligase activity in liver cancer. Cancer Lett.

[CR40] Yang S, Chen Y, Sun F, Ni Q, Wang H, Huang Y, Zhang C, Liu K, Wang S, Qiu J (2016). Downregulated PIRH2 can decrease the proliferation of breast cancer cells. Arch Med Res.

[CR41] Yerlikaya A, Yontem M (2013). The significance of ubiquitin proteasome pathway in cancer development. Recent Pat Anticancer Drug Discov.

[CR42] Yin J, Zhu JM, Shen XZ (2015). The role and therapeutic implications of RING-finger E3 ubiquitin ligases in hepatocellular carcinoma. Int J Cancer.

[CR43] Zhang J, Wan L, Dai X, Sun Y, Wei W (2014). Functional characterization of Anaphase Promoting Complex/Cyclosome (APC/C) E3 ubiquitin ligases in tumorigenesis. Biochim Biophys Acta.

[CR44] Zhu R, Xi H, Li YH, Jiang H, Zou JF, Hou J (2009). Establishment of a bortezomib-resistant myeloma cell line and differential proteins analysis by MALDI-OF-MS. Zhejiang Da Xue Xue Bao Yi Xue Ban.

